# Association of prothrombin time, thrombin time and activated partial thromboplastin time levels with preeclampsia: a systematic review and meta-analysis

**DOI:** 10.1186/s12884-024-06543-7

**Published:** 2024-05-13

**Authors:** Ermiyas Alemayehu, Ousman Mohammed, Melaku Ashagrie Belete, Zewudu Mulatie, Habtu Debash, Alemu Gedefie, Daniel Gebretsadik Weldehanna, Bruktawit Eshetu, Agumas Shibabaw, Saba Gebremichael Tekele, Mihret Tilahun, Hussen Ebrahim

**Affiliations:** Department of Medical Laboratory Sciences, College of Medicine and Health Sciences, Wollo University, Dessie, Ethiopia

**Keywords:** Coagulation parameters, Hemostatic parameters, Prothrombin time, Thrombin time, Activated partial thromboplastin time, Preeclampsia

## Abstract

**Background:**

Preeclampsia (PE), an obstetric disorder, remains one of the leading causes of maternal and infant mortality worldwide. In individuals with PE, the coagulation-fibrinolytic system is believed to be among the most significantly impacted systems due to maternal inflammatory responses and immune dysfunction. Therefore, this systematic review and meta-analysis aimed to assess the association of prothrombin time (PT), thrombin time (TT) and activated partial thromboplastin time (APTT) levels with preeclampsia.

**Methods:**

This systematic review and meta-analysis was conducted in accordance with the PRISMA guidelines. Articles relevant to the study, published from July 26, 2013, to July 26, 2023, were systematically searched across various databases including PubMed, Scopus, Embase, and Hinari. The methodological quality of the articles was evaluated using the Joanna Briggs Institute critical appraisal checklist. Utilizing Stata version 14.0, a random-effects model was employed to estimate the pooled standardized mean difference (SMD) along with the respective 95% CIs. The I^2^ statistics and Cochrane Q test were utilized to assess heterogeneity, while subgroup analyses were performed to explore its sources. Furthermore, Egger’s regression test and funnel plot were employed to assess publication bias among the included studies.

**Results:**

A total of 30 articles, involving 5,964 individuals (2,883 with PE and 3,081 as normotensive pregnant mothers), were included in this study. The overall pooled SMD for PT, APTT, and TT between PE and normotensive pregnant mothers were 0.97 (95% CI: 0.65–1.29, *p* < 0.001), 1.05 (95% CI: 0.74–1.36, *p* < 0.001), and 0.30 (95% CI: -0.08-0.69, *p* = 0.11), respectively. The pooled SMD indicates a significant increase in PT and APTT levels among PE patients compared to normotensive pregnant mothers, while the increase in TT levels among PE patients was not statistically significant.

**Conclusions:**

The meta-analysis underscores the association between PE and prolonged PT and APTT. This suggests that evaluating coagulation parameters like PT, APTT, and TT in pregnant women could offer easily accessible and cost-effective clinical indicators for assessing PE. However, multicenter longitudinal studies are needed to evaluate their effectiveness across various gestational weeks of pregnancy.

**Supplementary Information:**

The online version contains supplementary material available at 10.1186/s12884-024-06543-7.

## Introduction

Preeclampsia (PE), an obstetric disorder, is categorized as mild PE if the patient’s blood pressure (BP) ranges from 140/90 to 160/110 mmHg and they exhibit proteinuria of ≥ 1, and as severe PE if BP is ≥ 160/110 mmHg, with proteinuria exceeding 3 + along with edema and other significant symptoms [1]. It affects approximately 2–5% of pregnancies globally, remaining one of the leading causes of maternal mortality worldwide [2, 3]. Each year, approximately 4 million women worldwide are diagnosed with PE. Sadly, this disease claims the lives of an estimated 76,000 women, along with half a million fetuses and neonates, on a global scale annually [3, 4].

Uncontrolled PE can result in maternal complications such as multi-organ failure, eclampsia, seizures, hemorrhagic stroke, adult respiratory distress syndrome, HELLP syndrome (hemolysis, elevated liver enzymes, and low platelet count), placental abruption, disseminated intravascular coagulation (DIC), renal failure, and pulmonary edema. Neonatal complications may encompass intrauterine growth retardation, prematurity, and mortality [5–9]. In clinical practice, achieving precise diagnosis and consistently predicting PE has proven to be a challenging issue [10].

The pathophysiological changes observed in PE patients stem from vascular endothelial injury [11]. Placental and immunologic irregularities prompt the release of inflammatory cytokines which trigger inflammatory responses, vascular endothelial injury, and the exposure of collagen and tissue factors beneath the endothelium. Consequently, alterations occurring in the hemostasis system. This sequence of events can lead to fetal demise in utero, dysontogenesis, and various adverse obstetrical outcomes [12, 13].

Assessing the status of hemostasis, coagulation, and fibrinolysis systems in PE patients is crucial for determining disease severity, facilitating early clinical intervention, and improving the prognosis of maternal and infant health conditions [14]. In clinical practice, commonly utilized laboratory tests for assessing hemostasis, coagulation, and fibrinolysis function include prothrombin time (PT), activated partial thromboplastin time (APTT), international normalized ratio (INR), thrombin time (TT), antithrombin (AT), platelet count (PLT), mean platelet volume (MPV), among others [15, 16].

The APTT evaluates endogenous blood coagulation activity, specifically targeting factors IX, XI, and XII, and it is indispensable for monitoring heparin dosage [17, 18]. Meanwhile, TT measures clot formation duration and the conversion of fibrinogen to fibrin, playing a crucial role in diagnosing clotting disorders and monitoring heparin therapy [19, 20]. On the other hand, PT examines both extrinsic and common coagulation pathways, assisting in the detection of deficiencies in factors II, V, VII, and X, as well as low fibrinogen concentrations [21].

Numerous investigations have examined PT, APTT, and TT levels in individuals with PE, suggesting their potential as PE risk indicators. However, findings regarding the correlation between PT, APTT, TT levels, and PE risk have been contradictory. While some studies indicate reduced levels in PE patients [10, 22, 23], others suggest elevated levels [24, 25]. To resolve this inconsistency, we conducted a comprehensive meta-analysis comparing PT, APTT, and TT levels between PE patients and normotensive pregnant women, seeking to clarify their significant association with PE risk.

## Methods

### Protocol registration

The research adhered to the guidelines outlined in the Preferred Reporting Items for Systematic Reviews and Meta-analysis (PRISMA) statement (Supplementary Table **1**) [26]. The study protocol was registered on the PROSPERO under the registration number CRD42023448949.

### Literature search

A comprehensive search strategy was applied to retrieve studies reporting levels of PT, TT and APTT in PE patients and normotensive pregnant mothers. Literature searches were carried out systematically. Electronic databases like PubMed, Scopus, Embase, and Hinari were used. In addition, bibliographies of the identified studies were screened intentionally to include additional relevant studies omitted during electronic database searches. Keywords including “coagulation parameters” OR “coagulation profile” OR “coagulation abnormalities” OR “hemostatic parameters” OR “prothrombin time” OR “thrombin time” OR “activated partial thromboplastin time” AND “preeclampsia” OR “pregnancy induced hypertension” OR “complicated pregnancy” were searched. The last search was conducted on September 1, 2023. After literature search, all records were imported into the EndNote 20 software, and duplicate studies were removed. The detailed search strategies are shown in Supplementary Table **2**.

### Eligibility criteria

The inclusion criteria included the following: (1) studies that reported levels of PT, TT and APTT in both PE patients and normotensive pregnant mothers; (2) observational studies (cross-sectional, case-control, and cohort); and [3] studies published from July 26, 2013, to July 26, 2023. The exclusion criteria included [1] studies without reporting coagulation parameters; and [2] review articles, case reports, narrative reviews, conference abstracts without full information, editorials, commentaries, letters to the editor, and author replies.

### Data screening, extraction and quality assessment

The screening process was carried out by two independent reviewers (EA and HE). Literatures were initially excluded by screening the title and abstract. The remaining full texts were thoroughly scanned according to the eligibility criteria. Any disagreements between the reviewers were resolved by the involvement of a third reviewer (MAB). After selection of the included studies, the following data were extracted by two independent reviewers (AG and HD): first author, publication year, country, study design, sample size (PE and normotensive pregnant mothers), PE severity and levels of PT, TT and APTT. Continuous variables were presented as mean ± SD. For studies that reported only median and interquartile range (IQR), the Microsoft Excel software was used to convert them in to the form of mean ± SD as recommended by Wan et al. [27].

The quality of the studies was assessed using the Joana Brigg’s institute (JBI) tool [28] by two independent reviewers (ZM and DGW). The tool consists different items to assess the internal and external validity of cross-sectional studies, case-control studies, and cohort studies. Each item was assessed as either yes, no, unclear, or not available. Any discrepancies in the rating of the studies were resolved through discussions among the authors and when the discrepancies continue after discussion, a third person (OM) was involved to solve the discrepancies. Total scores ranged between 0 and 9, and studies with an average score of 50% and above were included in this meta-analysis study.

### Outcomes of interest

The primary outcome of interest was to determine the pooled standardized mean difference (SMD) of PT, APTT, and TT between PE patients and normotensive pregnant mothers.

### Data analysis

The data were analyzed using Stata 14.0 software. Cochran’s Q test and I^2^ statistics were used to assess the heterogeneity of the studies. The occurrence of significant heterogeneity was described as I^2^ test statistics values > 50% and Q test and its corresponding p-value < 0. 05 [29]. A random effect model was used to estimate the pooled SMD along with its 95% confidence interval between patients with PE and normotensive pregnant mothers. The results were presented using a forest plot. Subgroup analysis was conducted based on different factors like publication year, continent, study approaches, and PE severity to explore the potential sources of heterogeneity. Sensitivity analysis was conducted to identify disproportionately influencing the results. Moreover, publication bias was assessed using by checking of symmetry of the funnel plot and the Egger weighted regression method. Asymmetry of the funnel plot [30] and *p* < 0.05 from the Egger test statistics were considered suggestive of statistically significant publication bias.

## Results

### Literature search results and study selection process

**Fig. 1:**
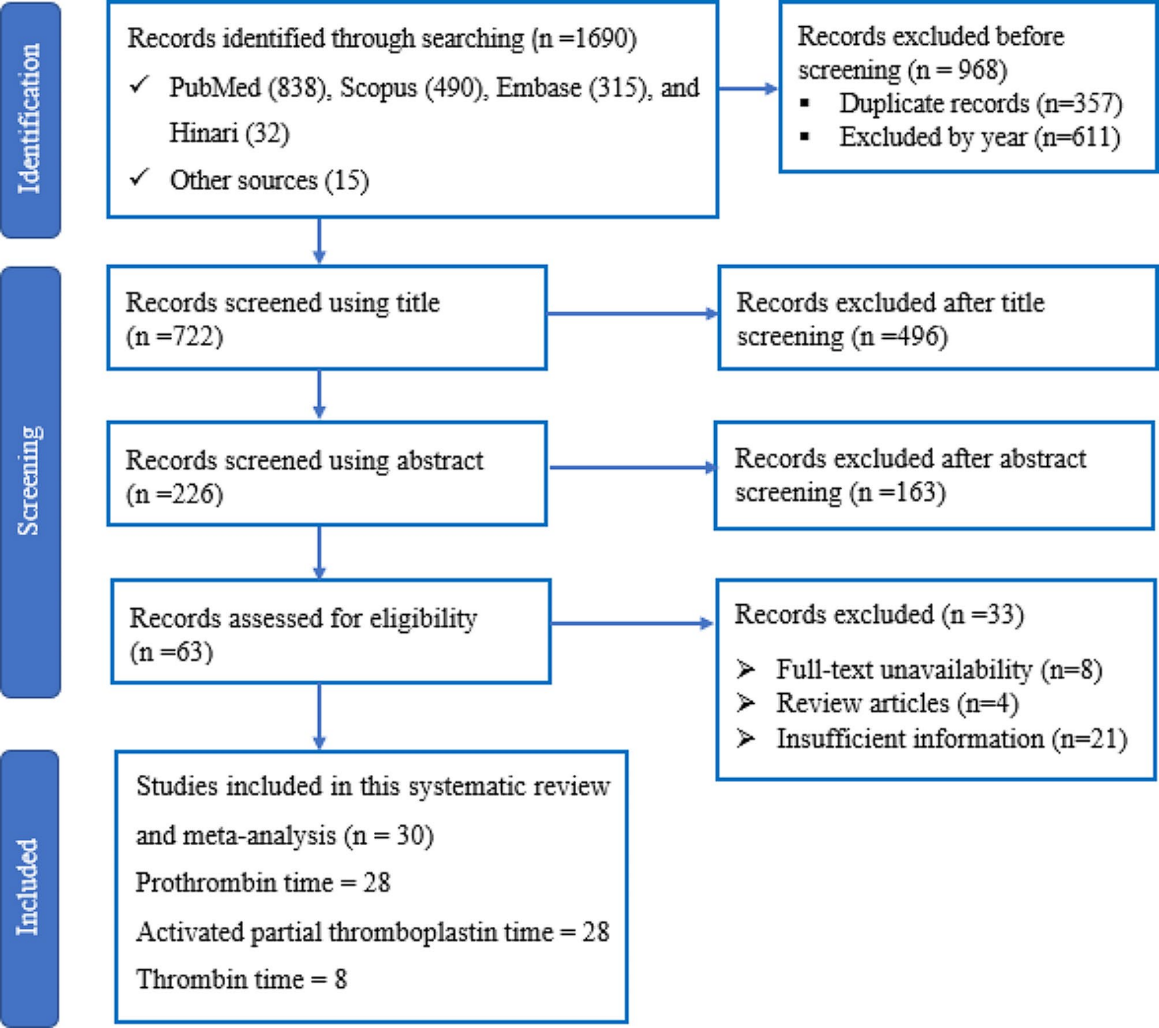
Flow chart describing the process of selecting studies for this meta-analysis

The initial search yielded 1690 results, comprising 838 from PubMed, 490 from Scopus, 315 from Embase, 32 from Hinari, and 15 from other sources. Following the removal of duplicate entries (357) and the exclusion of studies by year (611), a total of 722 studies were retained. The screening of titles and abstracts resulted in 63 studies, and upon a thorough examination of the full texts, 33 studies were excluded for various reasons. Ultimately, 30 studies were included in this meta-analysis. Figure 1 provides a comprehensive overview of the search process and the reasons for exclusion.

### Characteristics of the included studies

In this meta-analysis, a total of 30 studies were included, spanning various regions and publication years from 2014 to 2023. Among these, 10 studies originated from China [10, 22–25, 31–35], 11 from India [36–46], 2 from Pakistan [47, 48], 2 from Nigeria [49, 50], and 1 each from France [51], Iraq [52], Italy [53], Russia [54], and Sudan [55]. In total, 5,964 individuals participated across these studies, with 2,883 identified as patients with preeclampsia and 3,081 as normotensive pregnant mothers. From the total preeclampsia patients, Among the 30 articles, 28 reported PT, 28 reported APTT, and 8 reported TT levels. The baseline characteristics of the included studies are summarized in Table 1.

### Methodological quality and risk of bias assessment

The quality of each study was assessed using the JBI quality assessment tool and the results of this assessment are recorded in Supplementary Table 3. Overall, the studies showed a high‑quality methodology of conduction, indicating that they do not carry a high risk of biased results.

**Table 1:** Characteristics of the included studies in the pooled SMD estimate of PT, APTT and TT

Authors	Year	County	Study Design	N*o* of PE	N*o* of controls	PT levels		APTT levels		TT levels		PE Severity
						Mean ± SD of PE	Mean ± SD of controls	Mean ± SD of PE	Mean ± SD of controls	Mean ± SD of PE	Mean ± SD of controls	
Han et al. [23]	2014	China	R. case-control	41	79	NR	NR	27.30 **±** 2.40	28.90 **±** 2.70	12.90 **±** 0.60	14.10 **±** 1.30	Severe PE
Chen et al. [22]	2017	China	Retrospective	125	188	10.27 **±** 0.83	10.56 **±** 0.75	25.11 **±** 2.89	26.70 **±** 2.70	NR	NR	Un-defined
Chen et al. [31]	2019	China	Retrospective	128	128	11.39 **±** 2.43	11.42 **±** 0.83	29.86 **±** 4.75	31.03 **±** 18.67	16.60 **±** 1.15	16.13 **±** 1.12	Un-defined
Han et al. [25]	2019	China	R. case-control	72	72	11.48 **±** 6.99	11.07 **±** 0.65	NR	NR	16.45 **±** 1.40	16.25 **±** 0.86	Un-defined
Lidan et al. [32]	2019	China	NR	32	59	10.77 **±** 0.66	10.85 **±** 0.75	27.30 **±** 3.11	26.92 **±** 2.58	15.36 **±** 2.12	14.90 **±** 0.70	Mild PE
Lidan et al. [32]	2019	China	NR	26	59	10.57 **±** 0.62	10.85 **±** 0.75	26.15 **±** 4.40	26.92 **±** 2.58	15.51 **±** 1.10	14.90 **±** 0.70	Severe PE
Danyu et al. [33]	2019	China	Cross-sectional	48	100	12.52 **±** 0.75	12.74 **±** 0.62	25.39 **±** 2.21	25.21 **±** 1.94	NR	NR	Mild PE
Danyu et al. [33]	2019	China	Cross-sectional	131	100	12.17 **±** 0.85	12.74 **±** 0.62	26.83 **±** 2.71	25.21 **±** 1.94	NR	NR	Severe PE
Xu et al. [34]	2021	China	Cross-sectional	290	256	9.71 **±** 0.61	10.50 **±** 0.60	28.49 **±** 2.95	27.17 **±** 1.90	15.13 **±** 0.75	14.33 **±** 0.36	Un-defined
Cui et al. [24]	2023	China	Retrospective	28	161	12.25 **±** 1.41	12.19 **±** 0.72	32.43 **±** 4.43	31.31 **±** 3.10	15.74 **±** 1.53	15.10 **±** 1.00	Mild PE
Cui et al. [24]	2023	China	Retrospective	131	161	11.90 **±** 0.78	12.19 **±** 0.72	32.38 **±** 6.48	31.31 **±** 3.10	15.67 **±** 1.68	15.10 **±** 1.00	Severe PE
Jin et al. [35]	2023	China	Case-control	322	531	10.43 **±** 0.61	10.40 **±** 0.61	25.73 **±** 2.13	26.84 **±** 8.98	17.07 **±** 1.17	17.76 **±** 6.66	Un-defined
Ren et al. [10]	2023	China	Retrospective	61	73	11.04 **±** 0.97	11.85 **±** 3.49	28.52 **±** 3.19	28.74 **±** 3.80	NR	NR	Mild PE
Ren et al. [10]	2023	China	Retrospective	103	73	11.23 **±** 1.01	11.85 **±** 3.49	29.31 **±** 5.08	28.74 **±** 3.80	NR	NR	Severe PE
Lefkou et al. [51]	2020	France	R. case-control	34	35	13.24 **±** 0.80	12.23 **±** 0.59	32.64 **±** 1.83	29.53 **±** 1.62	NR	NR	Mild PE
Lefkou et al. [51]	2020	France	R. case-control	15	35	14.77 **±** 0.96	12.23 **±** 0.59	35.59 **±** 1.53	29.53 **±** 1.62	NR	NR	Severe PE
Chauhan et al. [36]	2014	India	Cross-sectional	59	100	13.78 **±** 1.82	13.58 **±** 1.08	29.50 **±** 1.78	29.31 **±** 3.39	NR	NR	Mild PE
Chauhan et al. [36]	2014	India	Cross-sectional	22	100	13.83 **±** 1.82	13.58 **±** 1.08	30.80 **±** 1.62	29.31 **±** 3.39	NR	NR	Severe PE
Anuradha [37]	2015	India	Case-control	40	40	17.80 **±** 1.11	17.10 **±** 0.98	NR	NR	NR	NR	Un-defined
Chaudhary et al. [38]	2016	India	Cross-sectional	40	100	14.23 **±** 0.98	12.70 **±** 0.96	29.43 **±** 1.92	26.58 **±** 1.88	NR	NR	Mild PE
Chaudhary et al. [38]	2016	India	Cross-sectional	35	100	16.59 **±** 3.62	12.70 **±** 0.96	32.48 **±** 5.01	26.58 **±** 1.88	NR	NR	Severe PE
Chaware et al. [39]	2017	India	Cross-sectional	60	120	13.87 **±** 1.02	13.74 **±** 1.19	28.56 **±** 2.56	28.23 **±** 2.35	NR	NR	Mild PE
Chaware et al. [39]	2017	India	Cross-sectional	30	120	14.22 **±** 1.11	13.74 **±** 1.19	30.80 **±** 6.01	28.23 **±** 2.35	NR	NR	Severe PE
Bhavana et al. [40]	2018	India	Case-control	78	150	12.48 **±** 1.08	11.97 **±** 0.31	32.08 **±** 2.93	30.54 **±** 1.35	NR	NR	Mild PE
Bhavana et al. [40]	2018	India	Case-control	32	150	13.85 **±** 3.70	11.97 **±** 0.31	34.73 **±** 5.72	30.54 **±** 1.35	NR	NR	Severe PE
Haldar et al. [41]	2020	India	Cross-sectional	30	30	14.81 **±** 1.02	13.80 **±** 1.10	29.73 **±** 2.77	26.66 **±** 2.44	NR	NR	Mild PE
Haldar et al. [41]	2020	India	Cross-sectional	30	30	15.75 **±** 1.61	13.80 **±** 1.10	31.29 **±** 3.71	26.66 **±** 2.44	NR	NR	Severe PE
Dundy et al. [42]	2020	India	NR	35	25	13.29 **±** 1.36	11.06 **±** 1.27	31.48 **±** 3.40	28.66 **±** 1.97	NR	NR	Mild PE
Dundy et al. [42]	2020	India	NR	35	25	13.95 **±** 0.99	11.06 **±** 1.27	35.44 **±** 3.31	28.66 **±** 1.97	NR	NR	Severe PE
Sharma et al. [43]	2021	India	Case-control	18	56	17.61 **±** 2.88	12.95 **±** 1.48	38.79 **±** 2.52	25.76 **±** 2.99	NR	NR	Mild PE
Sharma et al. [43]	2021	India	Case-control	6	56	18.88 **±** 1.12	12.95 **±** 1.48	41.85 **±** 1.95	25.76 **±** 2.99	NR	NR	Severe PE
Bhutani et al. [44]	2022	India	Cross-sectional	18	52	16.59 **±** 1.44	12.95 **±** 1.48	38.79 **±** 2.52	25.76 **±** 2.99	NR	NR	Mild PE
Bhutani et al. [44]	2022	India	Cross-sectional	6	52	17.61 **±** 2.88	12.95 **±** 1.48	41.85 **±** 0.01	25.76 **±** 2.99	NR	NR	Severe PE
Indora et al. [45]	2022	India	Case-control	36	50	13.86 **±** 1.84	11.23 **±** 1.25	31.52 **±** 7.81	27.84 **±** 4.64	NR	NR	Un-defined
Tadu et al. [46]	2023	India	Cross-sectional	25	50	10.45 **±** 1.00	11.12 **±** 1.49	27.18 **±** 3.28	29.34 **±** 4.11	NR	NR	Mild PE
Tadu et al. [46]	2023	India	Cross-sectional	25	50	10.44 **±** 1.06	11.12 **±** 1.49	26.59 **±** 2.66	29.34 **±** 4.11	NR	NR	Severe PE
Khan et al. [47]	2018	Pakistan	Cross-sectional	42	42	14.52 **±** 3.16	13.60 **±** 1.75	38.67 **±** 18.52	35.31 **±** 11.88	NR	NR	Un-defined
Shaheen et al. [48]	2020	Pakistan	Cross-sectional	73	100	12.88 **±** 0.37	12.10 **±** 0.23	34.31 **±** 0.82	31.54 **±** 0.52	NR	NR	Mild PE
Shaheen et al. [48]	2020	Pakistan	Cross-sectional	27	100	13.91 **±** 0.43	12.10 **±** 0.23	35.28 **±** 1.00	31.54 **±** 0.52	NR	NR	Severe PE
Ekun et al. [49]	2018	Nigeria	Cross-sectional	49	50	19.36 **±** 4.06	13.45 **±** 1.97	45.53 **±** 2.92	37.49 **±** 4.99	NR	NR	Un-defined
Oladosu-olayiwola et al. [50]	2018	Nigeria	Cross-sectional	85	85	13.90 **±** 1.00	14.10 **±** 0.98	35.40 **±** 4.80	34.40 **±** 1.90	NR	NR	Un-defined
Sami et al. [52]	2022	Iraq	Case-control	50	50	12.50 **±** 1.20	11.10 **±** 1.20	27.70 **±** 3.30	26.10 **±** 4.40	NR	NR	Un-defined
Spiezia et al. [53]	2015	Italy	Cross-sectional	30	60	11.00 **±** 0.11	10.30 **±** 0.16	26.00 **±** 3.00	28.00 **±** 3 0.00	NR	NR	Un-defined
Golovchenko et al. [54]	2018	Russia	Cross-sectional	250	209	NR	NR	32.33 **±** 5.96	37.83 **±** 4.85	15.00 **±** 2.98	14.33 **±** 2.98	Un-defined
Abass et al. [55]	2016	Sudan	Case-control	30	30	14.20 **±** 3.48	12.90 **±** 1.13	38.32 **±** 7.71	35.60 **±** 6.96	NR	NR	Un-defined

*Note* APTT: activated partial thromboplastin time; NR: not reported; PE: preeclampsia; PT: prothrombin time; R: retrospective; TT: thrombin time

### Levels of PT in preeclampsia patients vs. normotensive pregnant mothers

**Fig. 2:**
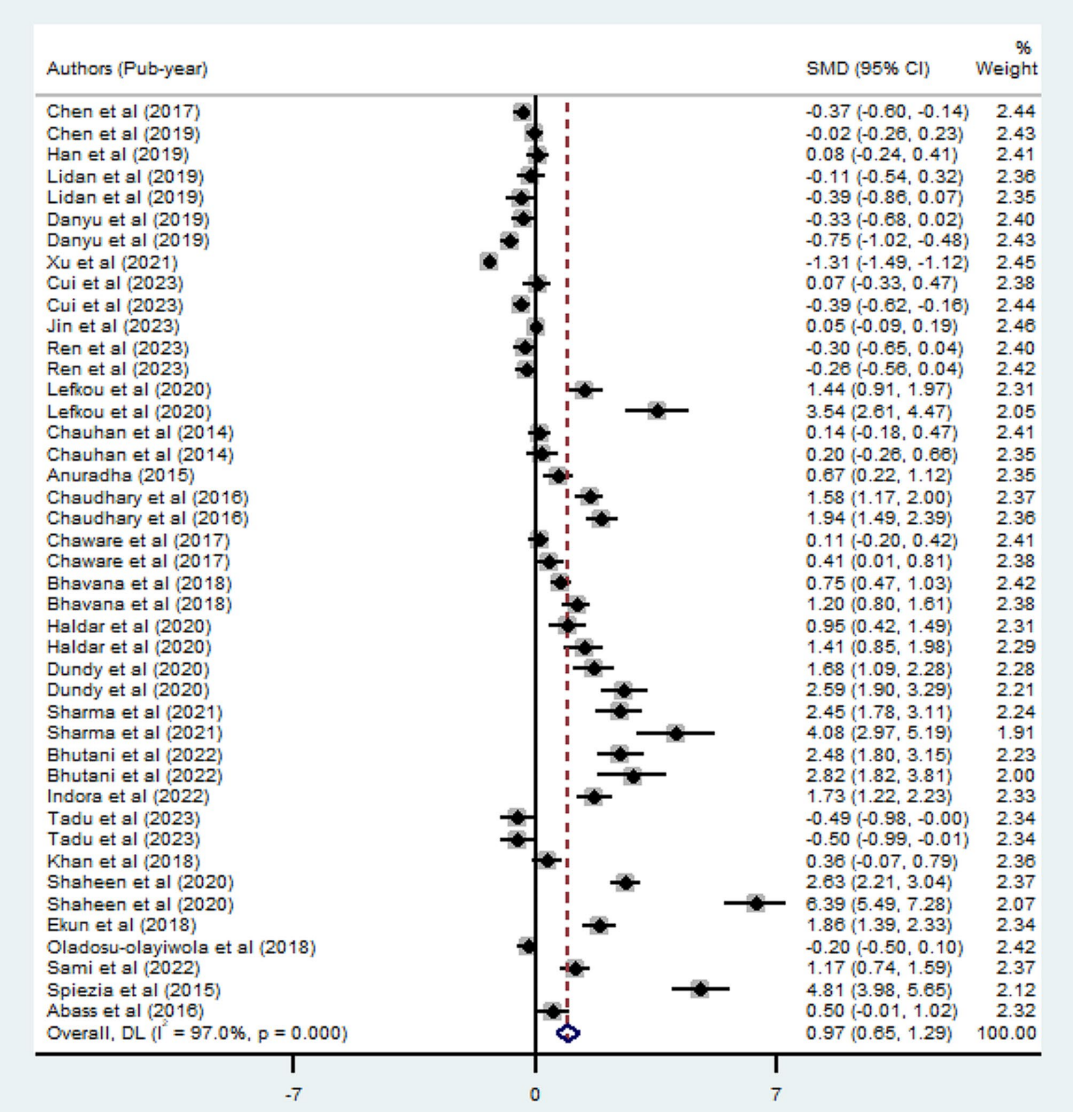
Forest plot of SMD of PT between preeclamptic and normotensive pregnant mothers

A random-effect model was employed to compare PT levels in preeclamptic and normotensive pregnant mothers, incorporating findings from twenty-eight studies. The results revealed a significant elevation in PT levels among patients with preeclampsia (SMD: 0.97; 95% CI; 0.65–1.29, *p* < 0.001). However, this association exhibited high heterogeneity, with an I^2^ value of 97%. The forest plot depicting the meta-analysis is presented in Fig. 2. Furthermore, using the random-effects model, the estimated pooled mean PT in preeclamptic mothers was 13.02 (95% CI; 12.38–13.66), whereas in normotensive pregnant mothers, it was 12.16 (95% CI; 11.75–12.57).

In the subgroup analysis based on publication year, it was found that the pooled SMD of PT was significantly higher in preeclamptic patients compared to normotensive pregnant mothers in studies conducted both between 2013 and 2018 (SMD: 0.88; 95% CI; 0.43–1.34, *p* = 0.002) and 2019–2023 (SMD: 1.02; 95% CI; 0.59–1.45, *p* < 0.001). Substantial heterogeneity was observed in both time periods, with I^2^ values of 95.5% and 97.5%, respectively. Also, the subgroup analysis based on continent revealed a significant increase in PT among preeclamptic patients in Asia (SMD: 1.02; 95% CI; 0.59–1.45, *p* < 0.001) and Europe (SMD: 1.02; 95% CI; 0.59–1.45, *p* < 0.001), accompanied by notable heterogeneity with I^2^ values of 96.9% and 95.9%, respectively. In addition, when considering the severity of PE, all subgroups (severe, mild, and undefined) showed a significantly increased PT compared to normotensive pregnant mothers, with pooled SMD values of 1.41 (95% CI; 0.70, 2.12, *p* < 0.001), 0.85 (95% CI; 0.35, 1.35, *p* = 0.001), and 0.66 (95% CI; 0.14, 1.18, *p* = 0.013), respectively. Significant heterogeneity was observed in all these subgroups, with I^2^ values of 97.4%, 95.5%, and 97.5%, respectively (Table 2).

### Levels of APTT in preeclampsia patients vs. normotensive pregnant mothers

**Fig. 3:**
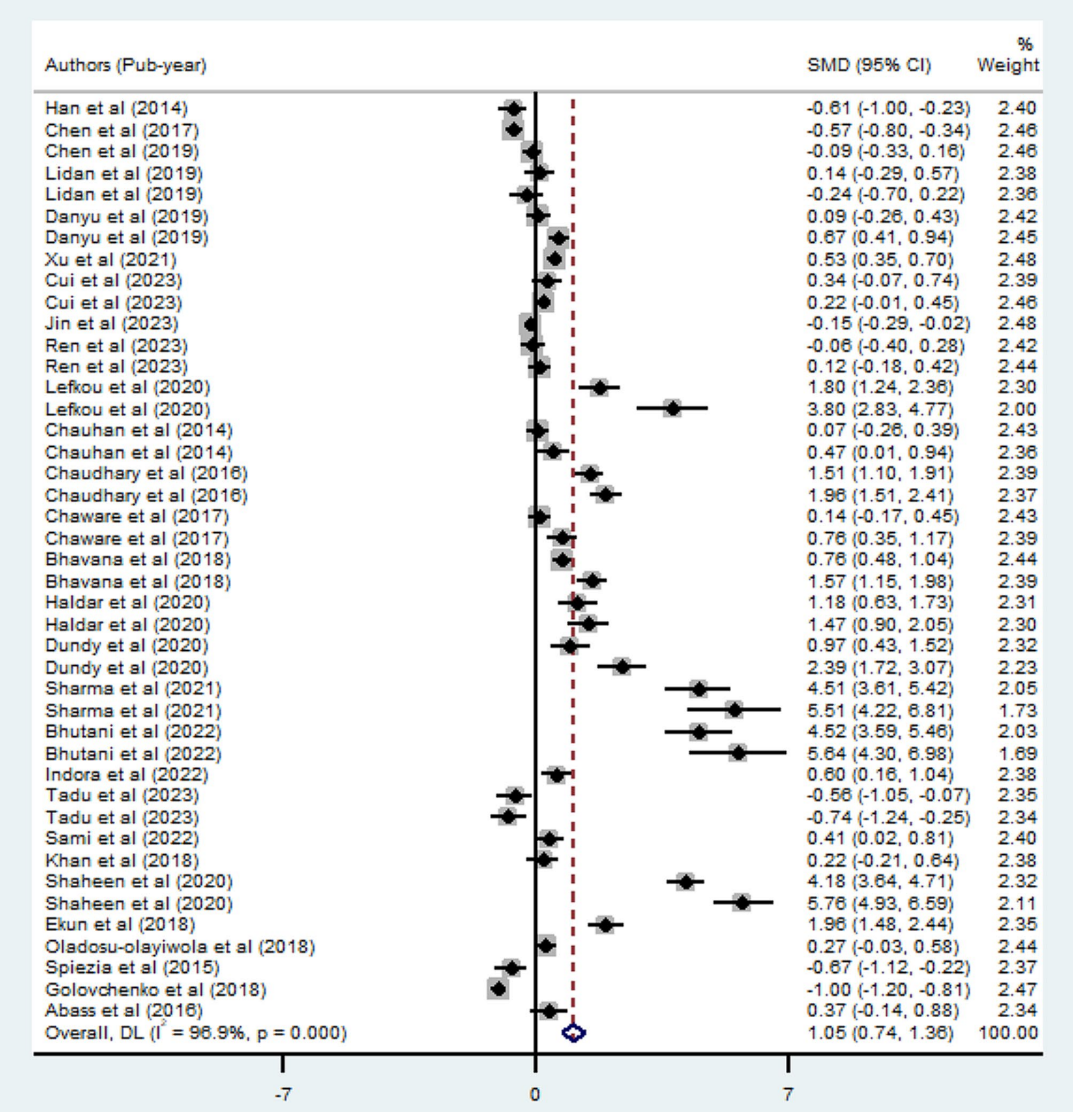
Forest plot of SMD of APTT between preeclamptic and normotensive pregnant mothers

A meta-analysis of twenty-eight studies, utilizing a random-effect model, indicated that the estimated combined mean value of APTT in pregnant mothers with preeclampsia was 31.70 (95% CI; 29.31–34.09), while it was 28.93 (95% CI; 28.06–29.80) in normotensive pregnant mothers. The overall pooled SMD analysis demonstrated a statistically significant elevation in APTT values among preeclamptic patients compared to normotensive pregnant mothers (SMD: 1.05; 95% CI; 0.74–1.36, *p* < 0.001). Notably, there was considerable heterogeneity among the included studies (I^2^ = 96.9%). The forest plot illustrating these findings is presented in Fig. 3.

Subgroup analysis, categorized by publication year, revealed a noteworthy elevation in the pooled SMD of APTT among preeclamptic patients compared to normotensive pregnant mothers in studies conducted between 2019 and 2023 (SMD: 1.57; 95% CI; 0.97–2.17, *p* < 0.001) with a substantial degree of heterogeneity (I^2^ = 97.1%). Furthermore, the subgroup analysis based on continent demonstrated a significant increase in APTT among preeclamptic patients in Asia (SMD: 1.19; 95% CI; 0.73–1.64, *p* < 0.001) with considerable heterogeneity (I^2^ = 98.5%), while no significant differences were observed in other continents. Additionally, the subgroup analysis, considering the severity of preeclampsia, indicated that both severe and mild preeclamptic patients exhibited a significantly elevated APTT compared to normotensive pregnant mothers, with pooled SMD values of 1.77 (95% CI; 0.91, 2.62, *p* < 0.001, I^2^ = 98.6%) and 1.30 (95% CI; 0.47, 2.12, *p* < 0.001, I^2^ = 98.6%), respectively (Table 2).

### Levels of TT in preeclampsia patients vs. normotensive pregnant mothers

**Fig. 4:**
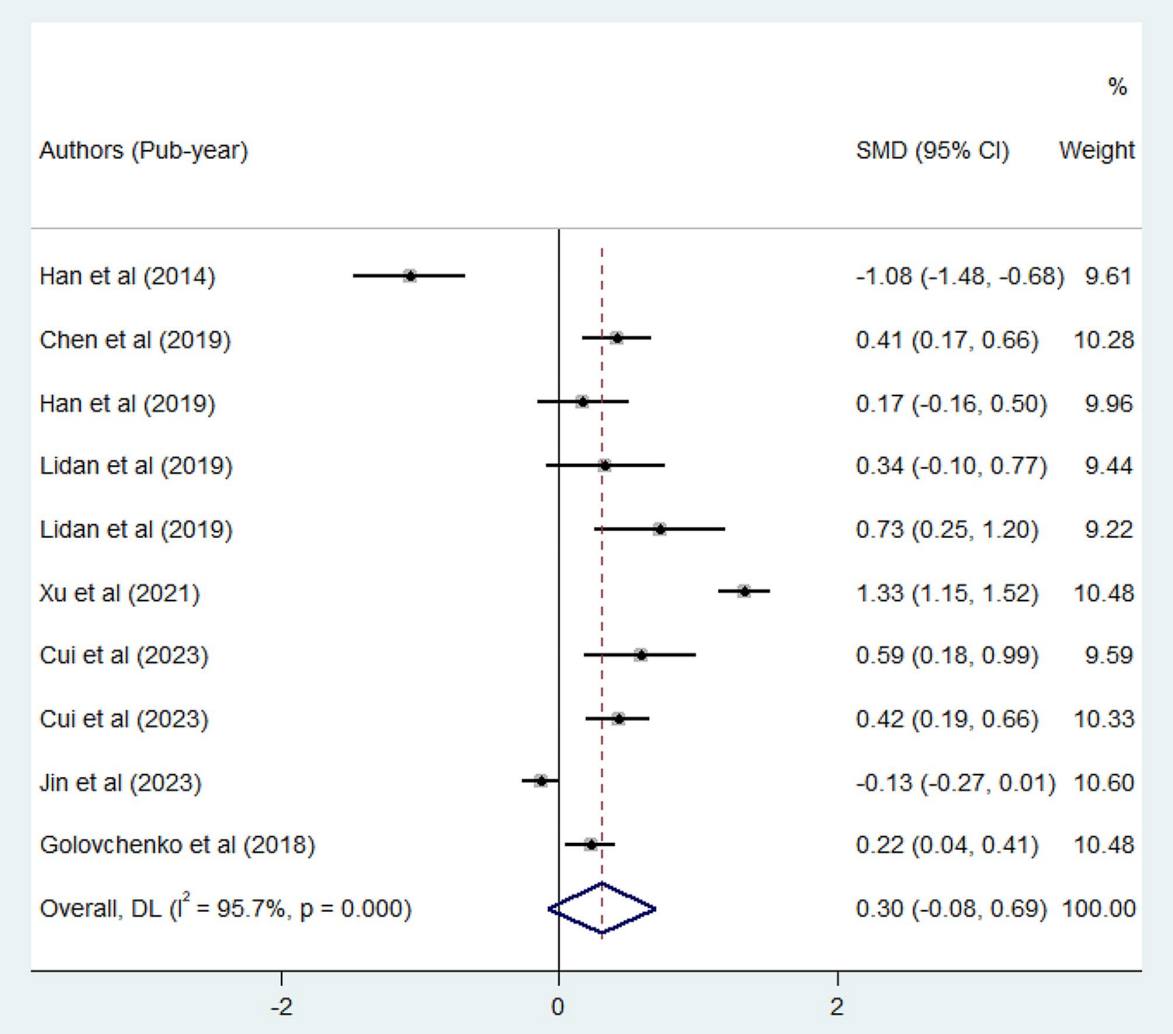
Forest plot of SMD of TT between preeclamptic and normotensive pregnant mothers

A random-effect meta-analysis was conducted on the pooled SMD for TT based on eight extracted studies. The overall pooled SMD revealed there is no significant difference in TT among preeclamptic patients compared to normotensive pregnant mothers (SMD: 0.30, 95% CI; -0.08-0.69, *p* = 0.11) as illustrated in Fig. 4. The estimated pooled mean of TT in preeclamptic mothers was 15.38 (95% CI; 14.29–16.48), while in normotensive pregnant mothers, it was 14.80 (95% CI; 14.32–15.28).

Subgroup analysis, considering the publication year, revealed a notable elevation in the pooled SMD of TT among preeclamptic patients compared to normotensive pregnant mothers in studies conducted between 2019 and 2023 (SMD: 0.48; 95% CI; 0.03–0.92, *p* = 0.02) with substantial heterogeneity (I^2^ = 96.1). Additionally, when examining preeclampsia severity, only mild preeclamptic patients displayed a significant increase in TT, evidenced by a pooled SMD value of 0.46 (95% CI; 0.17–0.76, *p* < 0.001) with no observed heterogeneity (I^2^ = 0.0%) (Table 2).

**Table 2:** Subgroup analysis of pooled SMD of PT, APTT and TT

**Parameters**	**Subgroup**		N*o* of studies	Pooled SMD (95% CI), *P*-value	Heterogeneity	
					I^2^	*P*-value
**PT**	Year	2013–2018	15	0.88 (0.43, 1.34), 0.002	95.4%	< 0.001
		2019–2023	28	1.02 (0.59, 1.45), < 0.001	97.5%	< 0.001
	Continent	Asia	37	0.82 (0.49, 1.15), < 0.001	96.9%	< 0.001
		Africa	3	0.71 (-0.53, 1.96), 0.263	96.2%	< 0.001
		Europe	3	3.24 (1.09, 5.40), < 0.001	95.9%	< 0.001
	Approaches	Prospective	30	1.18 (0.74, 1.62), < 0.001	97.6%	< 0.001
		Retrospective	9	0.27 (-0.13, 0.66), 0.183	92.5%	< 0.001
	PE severity	Un-defined	13	0.66 (0.14, 1.18), 0.013	97.5%	< 0.001
		Mild PE	15	0.85 (0.35, 1.35), 0.001	95.5%	< 0.001
		Severe PE	15	1.41 (0.70, 2.12), < 0.001	97.4%	< 0.001
**APTT**	Year	2013–2018	16	0.44 (-0.04, 0.93), 0.061	97.1%	< 0.001
		2019–2023	27	1.57 (0.97, 2.17), < 0.001	98.8%	< 0.001
	Continent	Asia	36	1.19 (0.73, 1.64), < 0.001	98.5%	< 0.001
		Africa	4	0.87 (-0.23, 1.97), 0.103	99.0%	< 0.001
		Europe	3	0.97 (-1.01, 2.95), 0.277	95.8%	< 0.001
	Approaches	Prospective	32	1.39 (0.86, 1.93), < 0.001	98.8%	< 0.001
		Retrospective	9	0.51 (-0.07, 1.09), 0.078	96.6%	< 0.001
	PE severity	Un-defined	13	0.14 (-0.24, 0.53), 0.464	96.4%	< 0.001
		Mild PE	15	1.30 (0.47, 2.12), < 0.001	98.6%	< 0.001
		Severe PE	15	1.77 (0.91, 2.62), < 0.001	98.6%	< 0.001
**TT**	Year	2013–2018	2	-0.41 (-1.69, 0.86), 0.525	97.3%	< 0.001
		2019–2023	8	0.48 (0.03, 0.92), 0.02	96.1%	< 0.001
	Approaches	Prospective	3	0.47 (-0.41, 1.3), 0.278	98.9%	< 0.001
		Retrospective	5	0.11 (-0.38, 0.60), 0.639	92.6%	< 0.001
	PE severity	Un-defined	5	0.40 (-0.17, 0.98), 0.157	97.2%	< 0.001
		Mild PE	2	0.46 (0.17, 0.76), < 0.001	0,0%	0.404
		Severe PE	3	0.02 (-1.00, 1.04), 0.964	96.2%	< 0.001

*Note* APTT: activated partial thromboplastin time; PE: preeclampsia; PT: prothrombin time; SMD: standardized mean difference; TT: thrombin time

### Publication Bias

**Fig. 5:**
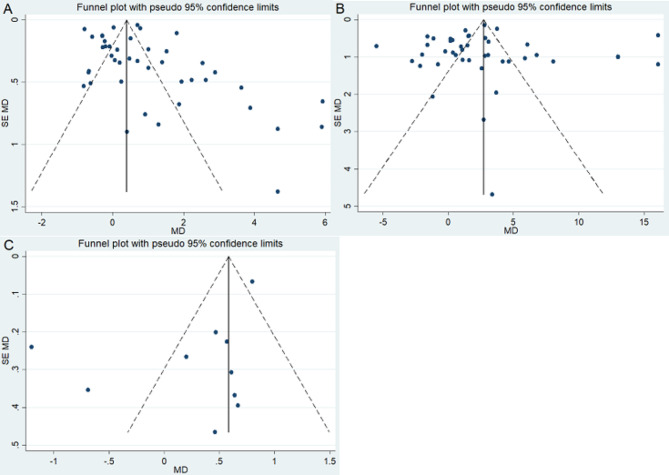
Assessment of publication bias for PT (**A**), APTT (**B**), and TT (**C**) levels

The assessment of included studies for publication bias utilized Egger’s test and the funnel plot. The results from Egger’s test indicated that the p values for all parameters (PT, APTT, and TT) were greater than 0.05. Furthermore, a visual inspection of the funnel plot revealed symmetry for all parameters. Both these outcomes suggest the absence of publication bias (Table 3and Fig. 5).

**Table 3:** Assessment of publication bias using Eger’s test statistics

Parameters	Std_Eff	Coef.	Std. Err.	T	*P*> |t|	95% CI
**PT**	Slope	0.2299972	0.1619281	1.42	0.163	− 0.0970232, 0.5570177
	Bias	1.596219	1.071991	1.49	0.144	− 0.5687096, 3.761148
**APTT**	Slope	2.805624	0.9606866	2.92	0.006	0.8654786, 4.74577
	Bias	− 0.2259826	1.779534	-0.13	0.900	-3.819825, 3.36786
**TT**	Slope	0.9270916	0.2349816	3.95	0.004	0.3852231, 1.46896
	Bias	-2.539793	1.367755	-1.86	0.100	-5.693842, 0.6142565

### Sensitivity analysis

**Fig. 6:**
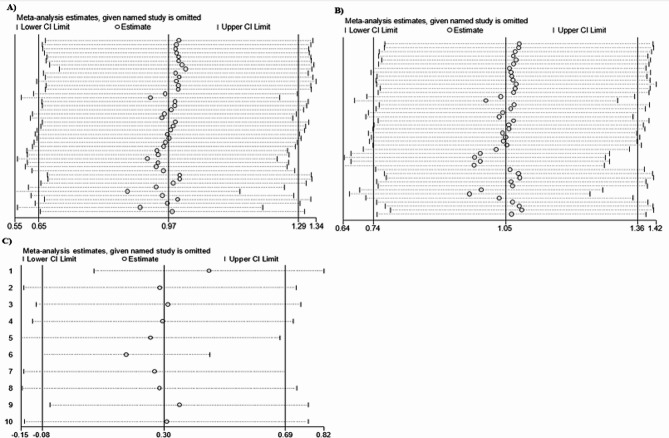
Sensitivity analysis assesses the impact of individual studies on meta-analytic results for PT (**A**), APTT (**B**), and TT (**C**), ensuring the robustness of findings

A sensitivity analysis was caried out using random effect models to assess the impact of individual studies on the combined SMD of PT, APTT, and TT levels in preeclamptic versus normotensive pregnant mothers. The results indicated that the exclusion of specific studies did not significantly affect the overall SMD of PT, APTT, and TT levels between the two groups (Fig. 6). This suggests that the results are robust and credible.

## Discussion

Preeclampsia, a significant complication in pregnancy, has remained a prominent global health concern for an extended duration, contributing significantly to perinatal and maternal mortality and morbidity worldwide [4, 44]. In patients with PE, maternal inflammatory responses and immune dysfunction significantly impact the coagulation-fibrinolytic system [56]. The heightened state of hypercoagulability in women with PE can result in systemic metabolic disorders and multiple organ dysfunction, posing a threat to both maternal and fetal well-being. Hence, the coagulative and fibrinolytic status serve as valuable predictors for the onset and clinical severity of PE [23]. Therefore, this systematic review and meta-analysis aimed to assess the SMD of PT, TT, and APTT between preeclampsia and normotensive pregnant mothers.

Our findings revealed a significant elevation in PT levels among patients with PE compared to normotensive pregnant mothers, with a pooled SMD of 0.97 (95% CI: 0.65–1.29, *p* < 0.001). Similarly, APTT levels in PE patients showed a noteworthy increase, as indicated by a pooled SMD of 1.05 (95% CI: 0.74–1.36, *p* < 0.001). Conversely, TT exhibited a slight increase in PE patients compared to normotensive pregnant mothers, but the difference did not reach statistical significance, with a pooled SMD of 0.30 (95% CI: -0.08-0.69, *p* = 0.11). These elevations in PT and APTT levels in PE patients stem from various factors. Endothelial dysfunction, a hallmark of PE [57], triggers the coagulation cascade, leading to a prothrombotic state and clot formation via both intrinsic and extrinsic pathways, prolonging APTT and PT. Additionally, impaired platelet function in PE [58] compromises clot formation efficiency, contributing to overall impaired coagulation and lengthening both clotting times. Moreover, systemic inflammation associated with PE [59] exacerbates coagulation cascade activation, intensifying clot formation and prolonging APTT and PT, worsening coagulation abnormalities in preeclampsia.

Due to significant heterogeneity among all three parameters, a subgroup analysis was undertaken using various covariates such as publication year, continent, and PE severity. In the subgroup analysis based on publication year, it was discovered that the pooled SMD of PT was significantly higher in preeclamptic patients compared to normotensive pregnant mothers in studies conducted both between 2013 and 2018 (SMD: 0.88; 95% CI; 0.43–1.34, *p* = 0.002) and 2019–2023 (SMD: 1.02; 95% CI; 0.59–1.45, *p* < 0.001). Regarding APTT levels, a noteworthy elevation was observed in the pooled SMD of APTT among preeclamptic patients compared to normotensive pregnant mothers in studies conducted between 2019 and 2023 (SMD: 1.57; 95% CI; 0.97–2.17, *p* < 0.001). Regarding TT levels, a notable elevation was found in the pooled SMD of TT among preeclamptic patients compared to normotensive pregnant mothers in studies conducted between 2019 and 2023 (SMD: 0.48; 95% CI; 0.03–0.92, *p* = 0.02). The results emphasize the variability of coagulation evaluation in preeclampsia studies, showcasing the changing patterns and varying focus on diverse coagulation measures over time. These results underscore the significance of continuous research and careful monitoring of coagulation profiles in individuals with preeclampsia to improve clinical care and prevent thrombotic complications linked to this condition.

In the subgroup analysis based on continent, a significant increase in PT among preeclamptic patients was observed in both Asia (SMD: 1.02; 95% CI; 0.59–1.45, *p* < 0.001) and Europe (SMD: 1.02; 95% CI; 0.59–1.45, *p* < 0.001). Regarding APTT levels, a significant increase was demonstrated among preeclamptic patients in Asia (SMD: 1.19; 95% CI; 0.73–1.64, *p* < 0.001), while no significant differences were observed in other continents. These findings highlight the importance of considering geographical factors when assessing and managing coagulation abnormalities in preeclampsia. Tailoring clinical approaches to address regional variations in coagulation profiles can enhance the precision and effectiveness of therapeutic interventions for preeclamptic patients worldwide. Additionally, further research into the underlying mechanisms driving regional differences in coagulation parameters may yield valuable insights into the pathophysiology of PE and inform targeted strategies for prevention and treatment on a global scale.

The subgroup analysis, considering the severity of PE, highlighted significant alterations in coagulation parameters among severe and mild preeclamptic patients compared to normotensive pregnant mothers. Both severe and mild cases exhibited notably elevated APTT levels relative to normotensive pregnant mothers, with pooled SMD values of 1.77 (95% CI; 0.91, 2.62, *p* < 0.001) and 1.30 (95% CI; 0.47, 2.12, *p* < 0.001), respectively, suggesting a consistent trend of prolonged clotting times across varying degrees of PE severity. Similarly, all subgroups - severe, mild, and undefined - displayed significantly increased PT levels compared to normotensive pregnant mothers, with pooled SMD values of 1.41 (95% CI; 0.70, 2.12, *p* < 0.001), 0.85 (95% CI; 0.35, 1.35, *p* = 0.001), and 0.66 (95% CI; 0.14, 1.18, *p* = 0.013), respectively, indicating widespread coagulation abnormalities in PE regardless of severity. PE, particularly in severe cases, can lead to DIC or a condition resembling DIC [60]. DIC is characterized by widespread activation of coagulation, which consumes clotting factors and platelets. As clotting factors are depleted, both APTT and PT can become prolonged due to the reduced availability of factors necessary for clot formation [61]. However, regarding TT, only mild preeclamptic patients showed a notable increase, supported by a pooled SMD value of 0.46 (95% CI; 0.17–0.76, *p* < 0.001) with no observed heterogeneity (I^2^ = 0.0%). All findings from the subgroup analysis based on PE severity underline the systemic impact of PE on coagulation profiles and highlight the importance of tailored management strategies based on the severity of the condition to effectively address associated risks.

The subgroup analyses based on publication year, severity of PE, and continent may not fully elucidate all underlying factors contributing to heterogeneity in the pooled estimate. Variations in study populations, including differences in demographic characteristics, gestational ages, management practices, and diagnostic criteria for PE across different regions, could introduce heterogeneity. Additionally, differences in assay techniques, laboratory protocols, and equipment calibration may contribute to variability in measured PT, APTT, and TT levels across studies, further impacting the pooled estimate. Unmeasured confounding variables, such as comorbidities, medication use, and lifestyle factors, could also influence study outcomes and contribute to heterogeneity in the pooled estimate.

The present review summarizes the SMD of PT, TT, and APTT levels between PE and normotensive pregnant mothers. Such analysis is essential for understanding the coagulation dynamics in these conditions. It not only provides a comprehensive overview of the differences in coagulation parameters but also aids in clinical decision-making and management strategies related to PE. In order to minimize selection bias, we performed a thorough literature search and incorporated studies that met clearly defined criteria. Additionally, the study adhered to the PRISMA guidelines and protocols during its implementation. While our findings highlight significant associations between PT, and APTT levels and PE, it is important to note that the blood samples were collected after the onset of the condition. Therefore, while these coagulation parameters may serve as potential markers for identifying PE, further research with blood samples collected prior to the onset of the condition is warranted to establish their predictive value. Nevertheless, these insights underscore the importance of continued investigation into the pathophysiological mechanisms underlying PE and the potential role of coagulation abnormalities in its development and progression.

However, the study is subject to potential limitations. Firstly, the articles included in this meta-analysis were predominantly from limited countries, which may introduce geographical bias. Another limitation pertains to the dynamic nature of coagulation parameters in PE. A comprehensive assessment of all coagulation parameters would offer vital diagnostic and prognostic insights. However, our analysis only focused on PT, APTT, and TT, while other coagulation parameters were not explored. Furthermore, a significant drawback of this study was the high level of heterogeneity observed across most analyses, stemming from differences in study populations, methodologies, and laboratory techniques, which could affect the validity and generalizability of the findings. Despite conducting subgroup analyses, the heterogeneity persisted, indicating that potential confounding factors influencing the association between coagulation parameters and PE may not have been fully considered in this meta-analysis.

### Conclusion and recommendations

The present meta-analysis highlights the association between PE and prolonged PT and APTT, suggesting that assessing these coagulation parameters in pregnant women could serve as readily accessible, cost-effective clinical indicators for evaluating PE. Moreover, these indices could offer reliable information for evaluating the severity of the disease and providing insight into the possible pathophysiology of PE. Policy-makers may consider integrating PT and APTT tests into routine prenatal screening protocols for all pregnant individuals, particularly those at high risk for PE. These tests can provide valuable information about coagulation status and help identify individuals who may require closer monitoring or early intervention. Additionally, given the observed significant elevation in PT and APTT levels among preeclamptic patients compared to normotensive pregnant mothers, healthcare systems may need to allocate resources for additional laboratory tests and monitoring to manage PE effectively. Furthermore, policy initiatives should focus on raising awareness among healthcare professionals and pregnant women about the importance of monitoring these coagulation parameters during pregnancy, particularly in the context of PE. Additionally, the diagnostic and prognostic capabilities of these parameters need exploration to facilitate early diagnosis and prognosis assessment of PE. Multicenter longitudinal studies are necessary to assess their utility across different gestational weeks of pregnancy.

### Electronic supplementary material

Below is the link to the electronic supplementary material.

Supplementary Material 1

Supplementary Material 2

Supplementary Material 3

## Data Availability

All necessary data for this systematic review and meta-analysis are available within the manuscript and its supporting information.
