# Supplementary material for: Association of prothrombin time, thrombin time and activated partial thromboplastin time levels with preeclampsia: a systematic review and meta-analysis
Source: BMC Pregnancy Childbirth. 2024 May 13;24:354. doi: 10.1186/s12884-024-06543-7 (PMC11092070; doi:10.1186/s12884-024-06543-7)
Supplement: Supplementary file 2 — Supplementary Material 2 [file 12884_2024_6543_MOESM2_ESM.docx]

| **SN** | **Databases** | **SEARCH TERM** | **No of Articles** | **Date** |
| --- | --- | --- | --- | --- |
| 1 | PubMed | ((((((("Coagulation parameters"[Title/Abstract]) OR ("coagulation profile"[Title/Abstract])) OR ("coagulation abnormalities"[Title/Abstract])) OR ("hemostatic parameters"[Title/Abstract])) OR ("prothrombin time"[Title/Abstract])) OR ("activated partial thromboplastin time"[Title/Abstract])) OR ("thrombin time"[Title/Abstract])) AND ("preeclampsia"[Title/Abstract]) OR ("complicated pregnancy"[Title/Abstract]) | 838 | 01/09  /2023 |
| 2 | Scopus | (TITLE-ABS-KEY ("coagulation parameters") OR TITLE-ABS-KEY ("coagulation profile") OR TITLE-ABS-KEY ("coagulation abnormalities") OR TITLE-ABS-KEY ("hemostatic parameters") OR TITLE-ABS-KEY (“prothrombin time”) OR TITLE-ABS-KEY ("activated partial thromboplastin time") OR TITLE-ABS-KEY ("thrombin time") AND TITLE-ABS-KEY (preeclampsia) OR TITLE-ABS-KEY ("pregnancy induced hypertension") OR TITLE-ABS-KEY ("complicated pregnancy")) | 490 | 01/09/  2023 |
| 3 | Embase | ('coagulation parameters':ti,ab,kw OR 'coagulation profile':ti,ab,kw OR 'coagulation abnormalities':ti,ab,kw OR 'hemostatic parameters':ti,ab,kw OR 'prothrombin time':ti,ab,kw OR 'thrombin time':ti,ab,kw OR 'activated partial thromboplastin time':ti,ab,kw) AND ('preeclampsia':ti,ab,kw OR 'pregnancy induced hypertension':ti,ab,kw OR 'complicated pregnancy':ti,ab,kw) | 315 | 01/09  /2023 |
| 4 | Hinari | ((Title:(coagulation parameters)) OR (Title:(coagulation profile)) OR (Title:(coagulation abnormalities)) OR (Title:(hemostatic parameters)) OR (Title:( prothrombin time)) OR (Title:( activated partial thromboplastin time)) OR (Title:( thrombin time))) AND ((Title:(preeclampsia)) OR (Title:(pregnancy induced hypertension)) OR (Title:(complicated pregnancy))) | 32 | 01/09  /2023 |
| 5 | Others | Use the titles that were identified from the bibliographies of articles selected from electronic database searches | 15 | 01-05/09  /2023 |

**Supplementary table 2** Searching strategy for association of prothrombin time, thrombin time and activated partial thromboplastin time levels with preeclampsia: A systematic review and meta-analysis
