# Supplementary material for: Association of prothrombin time, thrombin time and activated partial thromboplastin time levels with preeclampsia: a systematic review and meta-analysis
Source: BMC Pregnancy Childbirth. 2024 May 13;24:354. doi: 10.1186/s12884-024-06543-7 (PMC11092070; doi:10.1186/s12884-024-06543-7)
Supplement: Supplementary file 3 — Supplementary Material 3 [file 12884_2024_6543_MOESM3_ESM.docx]

**Supplementary table 3** Critical appraisal checklist for analytical cross-sectional studies for association of prothrombin time, thrombin time and activated partial thromboplastin time levels with preeclampsia: A systematic review and meta-analysis

| Author | Checklist criteria | | | | | | | |
| --- | --- | --- | --- | --- | --- | --- | --- | --- |
|  | Were the criteria for inclusion in the sample clearly defined? | Were the study subjects and the setting described in detail? | Was the exposure measured in a valid and reliable way? | Were objective, standard criteria used for measurement of the condition? | Were confounding factors identified? | Were strategies to deal with confounding factors stated? | Were the outcomes measured in a valid and reliable way? | Was appropriate statistical analysis used? |
| Xu et al | Yes | Yes | Yes | Yes | Unclear | Unclear | Yes | Yes |
| Danyu et al | Yes | Unclear | Yes | Yes | Unclear | Unclear | Yes | Yes |
| Ren et al | Yes | Yes | Yes | Yes | Yes | Yes | Yes | Yes |
| Lidan et al | Yes | Yes | Yes | Yes | Unclear | Unclear | Yes | Yes |
| Cui et al | Yes | Yes | Yes | Yes | Yes | Yes | Yes | Yes |
| Han L et al | Yes | Yes | Yes | Yes | Yes | Yes | Yes | Yes |
| Chen L et al | Yes | Yes | Yes | Yes | Yes | Yes | Yes | Yes |
| Jin et al | Yes | Yes | Yes | Yes | Yes | Yes | Yes | Yes |
| Han Q et al | Yes | Yes | Yes | Yes | Yes | Yes | Yes | Yes |
| Chen Y et al | Yes | Yes | Yes | Yes | Unclear | Unclear | Yes | Yes |
| Lefkou et al | Yes | Unclear | Yes | Yes | Unclear | Unclear | Yes | Yes |
| Haldar et al | Yes | Yes | Yes | Yes | No | No | Yes | Yes |
| Dundy et al | Yes | Yes | Yes | Yes | Unclear | Unclear | Yes | Yes |
| Bhutani et al | Yes | Yes | Yes | Yes | Unclear | Unclear | Yes | Yes |
| Indora et al | Yes | Yes | Yes | Yes | Unclear | Unclear | Yes | Unclear |
| Chauhan et al | Yes | Yes | Yes | Yes | No | No | Yes | Unclear |
| Anuradha | Yes | Yes | Yes | Yes | No | No | Yes | Yes |
| Sharma et al | Yes | Yes | Yes | Yes | Yes | Yes | Yes | Yes |
| Chaudhary et al | Yes | Yes | Yes | Yes | Unclear | Unclear | Yes | Unclear |
| Tadu et al | Yes | Yes | Yes | Yes | Unclear | Unclear | Yes | Yes |
| Chaware et al | Yes | Yes | Yes | Yes | Yes | Yes | Yes | Yes |
| Bhavana et al | Yes | Yes | Yes | Yes | Unclear | Unclear | Yes | Yes |
| Sami et al | Yes | Yes | Yes | Yes | Unclear | Unclear | Yes | Unclear |
| Spiezia et al | Yes | Yes | Yes | Yes | Unclear | Unclear | Yes | Yes |
| Ekun et al | Yes | Yes | Yes | Yes | Yes | Yes | Yes | Yes |
| Oladosu-olayiwola et al | Yes | Yes | Yes | Yes | No | No | Yes | Yes |
| Shaheen et al | Yes | Yes | Yes | Unclear | No | No | Yes | Yes |
| Khan et al | Yes | No | Yes | Unclear | No | No | Unclear | Unclear |
| Golovchenko et al | Yes | Yes | Yes | Yes | No | No | Yes | Yes |
| Abass et al | Yes | Yes | Yes | Yes | Unclear | Unclear | Yes | Yes |
